# Femtosecond Laser Single-Spot Welding of Sapphire/Invar Alloy

**DOI:** 10.3390/ma18163839

**Published:** 2025-08-15

**Authors:** Yuyang Chen, Yinzhi Fu, Xianshi Jia, Kai Li, Cong Wang

**Affiliations:** State Key Laboratory of Precision Manufacturing for Extreme Service Performance, College of Mechanical and Electrical Engineering, Central South University, Changsha 410083, China; 243711073@csu.edu.cn (Y.C.); f2254923917@163.com (Y.F.); likai01@csu.edu.cn (K.L.); wangcong@csu.edu.cn (C.W.)

**Keywords:** femtosecond laser, single-spot welding, ablation threshold, defect control, sapphire, Invar alloy

## Abstract

Ultrafast laser welding of glass/metal heterostructures has found extensive applications in sensors, medical devices, and optical systems. However, achieving high-stability, high-quality welds under non-optical contact conditions remains challenging due to severe internal damage within glass materials. This study addresses thermal management through synergistic control of thermal accumulation effects and material ablation thresholds. Using the sapphire/Invar alloy system as a model for glass/metal welding, we investigated thermal accumulation effects during ultrafast laser ablation of Invar alloy through theoretical simulations. Under a repetition rate of 1 MHz, the femtosecond laser raised the lattice equilibrium temperature by 700 K within 10 microseconds, demonstrating that high repetition rate femtosecond lasers can induce effective heat accumulation in Invar alloy. Furthermore, ablation thresholds for both materials were determined across varying repetition rates via the D^2^ method, with corresponding threshold curves systematically constructed. Finally, based on the simulation and ablation threshold calculation results, laser parameters were selected for ultrafast laser single point welding of sapphire and Invar alloy. The experimental results demonstrate effective thermal effect mitigation, achieving a maximum shear strength of 63.37 MPa. Comparative analysis against traditional scan welding further validates the superiority of our approach in thermal management. This work provides foundational theoretical and methodological guidance for ultrafast laser welding of glass/metal heterostructures.

## 1. Introduction

Sapphire is extensively utilized in high-power laser devices [1], in optical windows under extreme temperature and pressure conditions [2], and as substrate material for semiconductors and large-scale integrated circuits, owing to its exceptional optical properties, mechanical strength, and chemical stability [3,4]. These applications frequently necessitate joining sapphire to other structural or functional components, thereby requiring reliable sapphire-to-metal bonding solutions in manufacturing [5]. Invar alloy, which has a coefficient of thermal expansion (CTE) significantly lower than common metals, combined with low electrical conductivity, reduced thermal conductivity, and superior plasticity, toughness, and damage resistance, has been widely adopted in satellite waveguides, aerospace remote sensors, and precision instrumentation [6]. To minimize thermal mismatch-induced defects, Invar alloy exhibiting CTE compatibility with sapphire is strategically selected as the bonding material in practical applications [7].

Dissimilar materials, defined as pairs with substantially divergent physical and chemical properties, constitute over 90% of modern industrial components. Consequently, welding of dissimilar materials has emerged as a critical focus in joining technology. Glass/metal welding represents an urgently demanded category, where connectors combining impact-resistant and ductile metals with optical glass have become integral to diverse industries, including solar cells, medical devices, and sensor fabrication [6,8,9,10,11]. The ongoing miniaturization and expanding applications of these devices are driving increasingly stringent demands for precision micro-joining of glass and metal components.

The inherently low ductility and fracture toughness of glass materials render conventional joining methods such as arc welding, brazing, and friction welding inadequate for achieving high-quality glass/metal welding. In 2016, Xin et al. [12] deposited Ti/Mo bilayer metallic films onto sapphire using magnetron sputtering. High-temperature sintering was employed to ensure the weldability between sapphire and Kovar alloy, achieving a maximum joint strength of 69.6 ± 3.1 MPa. In 2019, Mu et al. [13] discovered that Ti significantly reduces the wetting angle of Sn-Ti alloy on sapphire. Utilizing Sn-3Ti alloy for vacuum brazing of sapphire at 600 °C, they obtained joints with a maximum shear strength of 30.3 MPa. In 2023, Yang et al. [14] adopted a novel pre-sintering approach to metallize the sapphire surface, followed by bonding sapphire to Ni-Ti metal using Au-Si filler metal at a bonding temperature of 460 °C, achieving a joint strength of 72 MPa. These improvements to traditional joining methods enable effective bonding between glass materials and metals. However, the aforementioned techniques require high welding temperatures, typically necessitate a vacuum environment, increase experimental costs, exacerbate residual thermal stresses within the weld seam, and suffer from drawbacks such as susceptibility to aging, complex procedures, and significant thermal stress. In recent years, the rapid advancement of laser technology has offered laser welding as a novel process for joining such dissimilar materials.

Although laser technology has become prevalent in modern industrial processing [15,16,17], precision welding involving glass remains a significant technical challenge. The translucency of glass limits direct energy absorption of most laser radiation, preventing sufficient energy deposition for material melting [18,19,20,21]. Furthermore, thermal effects associated with continuous-wave laser irradiation often induce severe thermal distortion and catastrophic fracture in brittle materials like sapphire. Consequently, traditional continuous-wave or long-pulse lasers are suboptimal for glass/metal welding applications [5].

Significant breakthroughs in laser pulse width reduction have enabled the widespread adoption of ultrashort pulse laser for glass/metal welding [22,23,24,25,26,27]. As the ultrashort-pulse laser category with the highest peak power and narrowest pulse width, femtosecond laser demonstrates exceptional promise for processing glass materials [28,29,30]. Currently, femtosecond laser welding research is typically categorized into two regimes: optical contact and non-optical contact. Optical contact refers to a condition where the specimen gap is smaller than λ/4. Performing femtosecond laser welding under this condition can significantly enhance weld quality [31,32,33]. In 2023, Pan et al. [7] successfully achieved direct bonding between sapphire and Fe-36Ni alloy for the first time using femtosecond laser microwelding technology. Owing to the highly localized femtosecond laser irradiation and minimal heat-affected zone, the resulting joint exhibited no porosity or microcracks, with a shear strength reaching 108.35 MPa. In 2024, Yang et al. [34] investigated surface roughness effects on shear strength during sapphire/Invar alloy welding using ultrashort pulses. Their results revealed a non-monotonic relationship: shear strength initially increased, then decreased with reducing surface roughness, peaking at 107.87 MPa when the arithmetic mean height (Sa) reached 0.131 μm. In 2025, Zhuo et al. [35] fabricated a robust sapphire–aluminum joint via direct ultrafast laser transmission microwelding. At a laser fluence of 0.56 J/cm^2^, the obtained joint was nearly defect-free, achieving a maximum room temperature shear strength of 128.1 ± 8.2 MPa. The aforementioned studies are all established on the basis of optical contact. Under optical contact conditions, achieving high-quality, high-stability glass/metal welding becomes considerably easier. However, the stringent requirements for surface roughness and flatness under optical contact conditions present significant barriers to industrial implementation of this technique.

Under non-optical contact conditions, the excessive specimen gap poses a significant challenge [34,36,37]. With insufficient laser energy, femtosecond laser welding induces nonlinear absorption and melting only within a small volume of material near the focal point. This localized melt pool is inadequate to bridge the large interfacial gap. Conversely, while excessive laser energy can fill the gap, it generates excessive thermal loading. This results in prohibitively high residual thermal stresses within the joint [38,39,40]. In both scenarios, the consequences are compromised joint strength and other associated issues. Achieving high-stability, high-quality welding under non-optical contact conditions remains a critical barrier to industrial implementation of ultrafast laser welding.

In 2008, Ozeki et al. [41] successfully welded glass to copper using femtosecond laser with clamping fixtures to provide pressure assistance. In 2014, Carter et al. [42] systematically analyzed and compared picosecond laser micro-spot welding of Al6082 with SiO_2_ and BK7. Their study employed pneumatic clamping fixtures to minimize interfacial gaps, thereby ensuring plasma formation during welding. Ultimately, they achieved Al6082-to-SiO_2_ joints with 13 MPa strength under non-optical contact conditions. In 2015, Zhang et al. [43] pioneered direct welding of alumino-silicate glass to metal using femtosecond laser without pressure assistance. However, their approach still required time-consuming mechanical polishing to achieve the necessary surface quality, a process that becomes prohibitively complex for large areas (hundreds of square millimeters or more). The aforementioned research primarily focuses on minimizing the interfacial gap between materials, either through auxiliary fixtures or surface pretreatment to enhance material surface quality. This approach aims to improve weld strength under non-optical contact conditions by reducing the gap. However, the use of complex fixtures and intricate pretreatment steps often entails high costs and low efficiency.

Controlling thermal effects during welding presents another viable approach for achieving high-quality, high-strength welds under non-optical contact conditions [44]. In 2023, Aeaby et al. [45] established and validated a Two-Temperature Model (TTM) to investigate the damage threshold characteristics of Au-based bimetallic films under femtosecond laser irradiation. Their work unraveled the influence of substrate materials and Au layer thickness on damage threshold behavior. Furthermore, they proposed an empirical function capable of accurately describing the non-monotonic variation of the damage threshold, providing a critical theoretical foundation for designing next-generation optical components. In 2024, Zhang et al. [46] utilized ultrashort-pulse laser microwelding technology to join sapphire and Invar alloy. They developed a predictive numerical model to calculate the interfacial temperature during laser irradiation, systematically investigating the relative contributions of thermal diffusion, thermal radiation, and heat accumulation during the welding process of these two materials. In 2025, Xu et al. [47] established a TTM for femtosecond laser ablation of alumina ceramics. This model was employed to study the heat accumulation mechanism during the interaction of femtosecond laser pulses with alumina ceramics in Burst mode. These theoretical studies provide valuable insights into the thermal phenomena occurring during ultrafast laser–matter interactions, establishing a solid theoretical foundation for controlling thermal effects in the welding process.

Currently, significant research efforts have been directed towards exploring methods to control thermal effects during welding [48,49,50]. In 2023, Zhang et al. [51] investigated the welding process of aluminum alloy to fused silica. Under low-energy conditions, insufficient diffusion capability of the aluminum alloy resulted in low joint strength. By increasing the femtosecond laser energy to 35 millijoules (mJ), they achieved tight bonding between the aluminum alloy and fused silica without special surface treatment, attaining a maximum shear strength of 7.77 MPa. In 2024, Yuan et al. [52] conducted pin welding experiments on alumina ceramics and titanium using femtosecond lasers. By employing a high-repetition rate, low-pulse energy femtosecond laser strategy, they enhanced the heat accumulation effect during welding. Ultimately, at a repetition rate of 1 MHz, they achieved exceptional performance, with a maximum joint strength of 134.9 MPa. These studies collectively demonstrate that controlling laser energy can effectively regulate thermal effects during the welding process, enabling high-quality welding under non-optical contact conditions. However, few studies have attempted to control welding thermal effects by manipulating the spatial distribution of laser energy, which will be the primary focus of this investigation.

This study address the control of thermal effects during welding through two primary approaches. The first is material threshold optimization: laser energy density must be controlled near the material’s ablation threshold to avoid internal glass damage. Studies demonstrate that the ratio of laser energy density to ablation threshold governs the welding mechanism [44]. Maintaining energy density slightly above the threshold enables adequate interfacial bonding between glass and metal while preventing structural degradation in the glass. The second approach is welding strategy modification: replacing conventional laser scan welding with single-spot welding reduces spatial energy concentration, thereby suppressing thermal accumulation effects.

To mitigate excessive thermal effects that compromise the structural integrity of glass materials during welding, this study employs a combined simulation experimental approach using sapphire/Invar alloy welding as a model system. Employing a combined simulation-experimental approach, we elucidated the energy accumulation mechanism of ultrafast lasers and obtained the ablation threshold curves for both sapphire and Invar alloy. Building upon this theoretical foundation, we further conducted experimental research on femtosecond laser single-spot welding of sapphire and Invar alloy. This investigation explored the influence of single-pulse energy and repetition rate on weld strength, aiming to minimize thermal effects during the welding process. During the research, we analyzed the results from dense single-spot welding and scanning welding. This analysis allowed us to elucidate the mechanism by which single-spot welding reduces thermal effects and enhances joint strength. Ultimately, by precisely controlling laser energy and reducing spatial energy concentration, we achieved high-quality, high-stability glass/metal welding under non-optical contact conditions.

## 2. Materials and Methods

**Figure 1:**
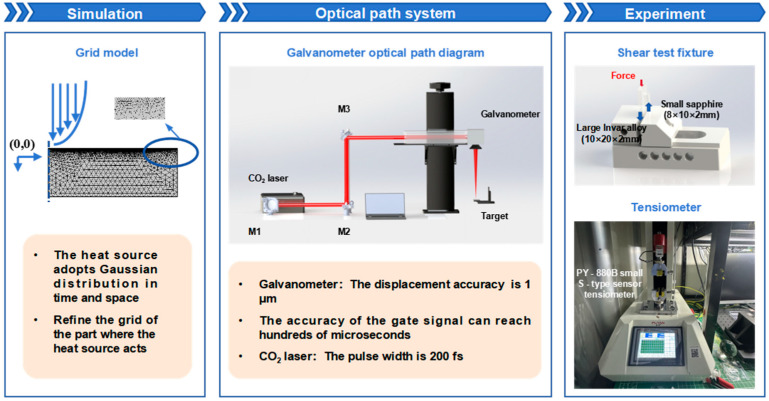
Schematic diagram schematically summarizing the research methodology for ultrafast laser single-spot welding of sapphire/Invar alloy. Simulation: Illustrates the thermal source configuration and mesh settings in the ultrafast laser ablation model of Invar alloy. Optical path system: Depicts the galvanometer scanning optical system employed in both ablation threshold testing and single-spot welding experiments, with detailed subfigures distinguishing these procedures. Experiment: Explains the equipment and methods used for shear strength testing.

This section details the research methodology, as shown in Figure 1. First, the theoretical foundation of the TTM for femtosecond laser ablation of Invar alloy is thoroughly introduced, followed by a presentation of the finite element model construction process. Subsequently, the sapphire and Invar alloy samples, along with the galvanometer scanning optical path system, required for the ablation threshold experiments and femtosecond laser single-spot welding experiments are introduced. Finally, the microstructural characterization techniques and the weld joint strength testing methods employed in this study are presented. Spanning from numerical simulation to process experiments, this research translates theoretical investigation into practical processing through a systematic, step-by-step approach, aiming to identify methods for mitigating thermal effects during glass/metal (dissimilar material) welding.

### 2.1. Physical Model

Femtosecond lasers exhibit extremely high peak power density. During interaction with matter, they primarily achieve material ablation through ionization mechanisms. This study establishes a physical model of the interaction between femtosecond laser and alumina ceramics based on the TTM. Using COMSOL Multiphysics 6.1 software, a partial differential equation (PDE) simulation model is developed to conduct numerical analyses for specific laser parameters.

In 1974, Soviet scholar Anisimov proposed the TTM, defined as a system of coupled PDEs. This framework comprises two interdependent PDEs that solve for the temperature distributions of electrons and lattices across spatial and temporal domains. We establish the TTM for femtosecond laser-Invar alloy interactions as follows [53]:

(1) $$C_{e}\frac{\partial T_{e}}{\partial t}=\frac{\partial }{\partial r}\left(k_{e}\frac{\partial T_{e}}{\partial x}\right)-G\left(T_{e}-T_{l}\right)+S\left(r,t\right),$$

(2) $$C_{l}\frac{\partial T_{l}}{\partial t}=G\left(T_{e}-T_{l}\right),$$

In these equations, $T_{e}$ and $T_{l}$ denote the electron and lattice temperatures, respectively; $C_{e}$ and $C_{l}$ represent the electron and lattice heat capacities; $k_{e}$ is the electron thermal conductivity; G stands for the electron-lattice coupling coefficient governing energy transfer between subsystems; Q (r,z,t) describes the laser heat source term; and r defines the radial distance in the ablation model of Invar alloy.

The electron heat capacity $C_{e}$ is a function of temperature, explicitly dependent on electron temperature. In the femtosecond laser ablation model for Invar alloy, this relationship can be expressed as [54].

(3) $$C_{e}(T_{e})=\left\{\begin{array}{lll} C_{e0}T_{e} & & T_{e}\leq \frac{T_{f}}{\pi^{2}}\\ \frac{2C_{e0}T_{e}}{3}+\frac{C_{e1}(T_{e})}{3} & & \frac{T_{f}}{\pi^{2}}<T_{e}\leq \frac{3T_{f}}{\pi^{2}}\\ N_{e}k_{B}+\frac{C_{e1}(T_{e})}{3} & & \frac{3T_{f}}{\pi^{2}}<T_{e}\leq T_{f}\\ \frac{3N_{e}k_{B}}{2} & & T_{e}>T_{f} \end{array}\right.,$$

(4) $$C_{e1}(T_{e})=\frac{C_{e0}T_{f}}{\pi^{2}}+\frac{3N_{e}k_{B}/2-C_{e0}T_{f}/\pi^{2}}{T_{f}-T_{f}/\pi^{2}}(T_{e}-T_{f}/\pi^{2}),$$

(5) $$C_{e0}=\frac{\pi^{2}N_{e}k_{B}}{2T_{f}},$$

In the equation, $N_{e}$ represents the free electron density in the material. For Invar alloy, this is estimated as 5.4 × 10^−16^ cm^−3^. The term $k_{B}$ denotes the Boltzmann constant, the value of $k_{B}$ is 1.380649 × 10^−23^ J· K^−1^, and $T_{f}$ is the Fermi temperature, taken as $T_{f}$ = 8.1353 × 10^4^ K.

$C_{l}$ is defined as the lattice specific heat capacity of a material. According to a study by Zheng et al. [55], the lattice specific heat can be assumed to be constant, with its magnitude approximating the material’s intrinsic specific heat. Based on the elemental composition of Invar alloy (64% Fe, 36% Ni), the volumetric heat capacity of Invar alloy is calculated as $C_{l}$ = 4.18 × 10^6^ J·m^−3^·K^−1^.

The electronic thermal conductivity $k_{e}$ can be obtained from Equation (6) [56]:

(6) $$k_{e}(T_{e},T_{l})=\frac{kT_{e}}{T_{l}},$$

In the formula, k represents the thermal conductivity of Invar alloy.

The electron–lattice coupling coefficient, governing energy transfer between electrons and the lattice, generally varies with their respective temperatures. According to Tang et al. [57], for TTM simulations of materials like iron and nickel, this coefficient can be treated as constant. Given that Invar alloy primarily consists of Fe (64%) and Ni (36%), we estimate its electron–lattice coupling coefficient G as 3.18 × 10^17^ W· m· K^−1^. This value is derived from the composition-weighted average of the Fe and Ni coefficients [56].

### 2.2. Finite Element Simulation

The governing equations are implemented using the built-in Coefficient Form PDE module in COMSOL Multiphysics software, with PDEs defined in their coefficient form:

(7) $$e_{a1}\frac{\partial^{2}Te}{\partial t^{2}}+d_{a1}\frac{\partial Te}{\partial t}+\nabla \cdot \left(-c_{1}\nabla Te-\alpha_{1}Te+\gamma_{1}\right)+\beta_{1}\cdot \nabla Te+a_{1}Te=f_{1},$$

(8) $$e_{a2}\frac{\partial^{2}Tl}{\partial t^{2}}+d_{a2}\frac{\partial Tl}{\partial t}+\nabla \cdot \left(-c_{2}\nabla Tl-\alpha_{2}Tl+\gamma_{2}\right)+\beta_{2}\cdot \nabla Te+a_{2}Te=f_{2},$$

(9) $$\nabla =\left[\frac{\partial }{\partial x},\frac{\partial }{\partial z}\right],$$

For the dual-temperature model established in this article, only the diffusion coefficient c, source term f, and damping coefficient $d_{a}$ need to be considered. Among them, for the electron temperature Equation (7), its source term includes the coupling term between the laser energy source Q (r,z,t) and the electron–lattice coupling term. For the lattice temperature Equation (8), the source term is the electron–lattice coupling term.

The expression for the laser energy source Q (r,z,t) is as follows:

(10) $$Q\;(r,z,t)=\frac{(1\;-\;R)F\delta_{0}}{t_{p}}\cdot Gaussianspace(r,z)\cdot Gaussiantime(t),$$

In the formula, R is the reflectivity of Invar alloy material; F is the laser energy density; $\delta_{0}$ is the absorption coefficient of the material; and $t_{p}$ is laser pulse width.

The distribution of laser energy source in both spatial and temporal domains adopts a Gaussian distribution, and its expressions Gaussianspace(r,z) and Gaussianspace(t) are as follows:

(11) $$Gaussianspace(r,z)=\exp(\;-\;2\frac{r^{2}}{r_{0}^{2}})\cdot \exp(\;-\;\frac{z}{\alpha }),$$

(12) $$Gaussianspace(t)=\exp(-4\ln2(\frac{t-2t_{p}}{t_{p}}{)}^{2}),$$

In the formula, Gaussianspace(r,z) and Gaussianspace(t) represent the spatial and temporal distribution of the laser, respectively; $r_{0}$ represents the radius of the laser beam.

The mesh setting of the model is shown in the Simulation section of Figure 1, with local refinement implemented in the laser irradiation zone. The boundary conditions of the model need to consider the difference between the simulation model and the actual processing environment. In order to better match the actual processing environment, the initial temperature of the electrons and lattice is set to 300 K. During the simulation process, the heat exchange between the model and the surrounding environment is not considered; only the conduction of laser energy from the processing area to other positions of the model is considered. Therefore, each surface of the model is set to zero flux. The material parameters of Invar alloy used in the numerical analysis are listed in Table 1.

### 2.3. Material Preparation and Laser Processing System

In this study, Invar alloy and sapphire are used as samples, with an Invar alloy size of 10 mm × 20 mm × 2 mm, a sapphire size of 10 mm × 20 mm × 2 mm used to study laser ablation threshold, and a sapphire size of 8 mm × 10 mm × 2 mm used to test laser welding strength. Prior to experiments, all samples are immersed in anhydrous ethanol and ultrasonically cleaned to remove contaminants. During experimentation, the sample surfaces must remain dry and clean.

The femtosecond laser processing system used in this experiment is shown in the Optical path system section of Figure 1. A commercial system (HR-Platform-0203, Wuhan Huaray Precision Laser Co., Ltd., Wuhan, China) is employed for ablation threshold testing and welding processing of the samples. This system emits pulses with a central wavelength of 1030 nm, a spot diameter of 30 μm, and a pulse duration of 238 fs. The femtosecond laser system offers a repetition rate adjustable from 50 kHz to 1000 kHz, with a maximum laser power of 54.6 W. The femtosecond laser beam is directed via several mirrors into a galvanometer scanner system. Integrated with this scanner system is an f-θ focusing lens (f = 80 mm) controlled by a computer. Samples are mounted on a motorized Z-axis translation stage, enabling flexible and precise focal plane adjustment.

This experiment aims to systematically investigate the ablation thresholds of Invar alloy and sapphire at different repetition rates and ablation durations. Based on the ablation threshold test results, laser parameters are selected to perform single-spot welding, to identify the optimal single-spot welding parameters. The laser parameters employed in this experiment are listed in Table 2.

### 2.4. Experiments and Characterization

An NM710 metallographic microscope (Nexcope, Ningbo, China) with magnification and measurement functions was used to observe the microscopic surface morphology of welded and tensile-fractured sapphire/Invar alloy joints. The system features wide-angle adjustable eyepieces providing 10× magnification, coupled with objectives offering magnifications of 5×, 10×, 20×, 50×, and 100×.

To determine the strength of the welded joint, a PY-880B small S-type sensor tensiometer (Puyan, China) was used for the shear test, as shown in Figure 1. To ensure uniform load distribution, a small sapphire piece was placed on a fixed platform, while a larger Invar alloy piece extended through the gap in this platform. The samples were positioned perpendicular to the platform surface without any tilt. During testing, the tensiometer gradually applies a load to the Invar alloy until separation occurred between the sapphire and Invar alloy. The specific experimental configuration is illustrated in the Experiment section of Figure 1.

## 3. Results and Discussions

### 3.1. Mechanism of Femtosecond Laser Thermal Ablation

This subsection will analyze the energy accumulation differences of ultrafast laser pulses with different repetition frequencies under the same total energy through numerical simulation. The model employed in this study is based on the TTM, where the electron subsystem and the lattice subsystem are mutually independent, yet interconnected. By analyzing the evolution of electron temperature and lattice temperature, we quantify the effects of single-pulse energy and repetition rate on the thermal accumulation phenomenon in Invar alloy. The findings will provide a critical reference for the subsequent optimization of experimental parameters.

**Figure 2:**
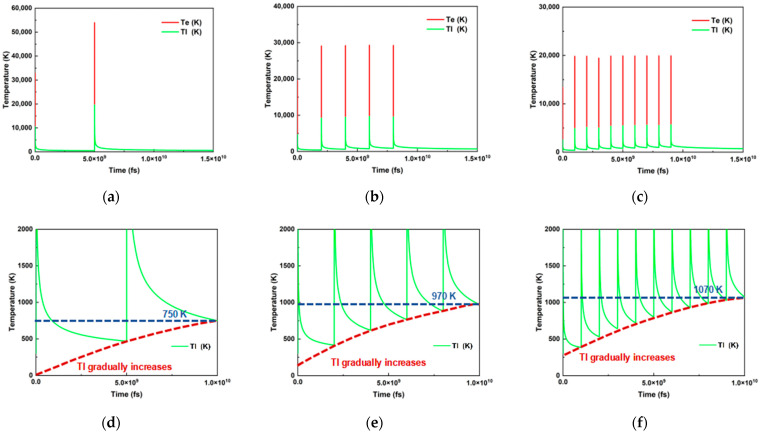
Numerical simulation results of femtosecond laser ablation of Invar alloy. (**a**–**c**) Temperature evolution during ablation using femtosecond laser at repetition rates of 200 kHz, 500 kHz, and 1000 kHz (30 W). (**d**–**f**) Schematic representations of pulse energy accumulation corresponding to 200 kHz, 500 kHz, and 1000 kHz repetition rates.

Numerical simulations reveal the dynamic process of ultrafast laser pulse energy accumulation. As shown in Figure 2a–c, ultrafast laser irradiation induces a sharp spike in electron temperature during the pulse. Following pulse termination, thermal energy in the electron subsystem gradually transfers to the lattice structure, resulting in a progressive rise in lattice temperature and a corresponding decline in electron temperature. Through coupling between the electron and lattice subsystems, thermal equilibrium between electrons and lattice is ultimately achieved, after which the system begins to cool. When subsequent pulses arrive, the electron temperature spikes again, and this cyclic energy transfer process repeats.

Figure 2 illustrates the influence of single pulse energy and repetition rate on temperature evolution. Since the total pulse energy remains constant, a decrease in repetition rate leads to a corresponding increase in single pulse energy. As shown in Figure 2a–c, at 200 kHz, the single pulse energy is five times higher than at 1000 kHz. Consequently, the highest thermal equilibrium temperature (20,000 K) was reached after the pulse at this rate. As the repetition rate increased and the single-pulse energy decreased, the achieved thermal equilibrium temperature progressively declined, to approximately 10,000 K at 500 kHz and 5000 K at 1000 kHz. While lower repetition rates yield higher thermal equilibrium temperatures, this does not imply greater overall accumulated heat. As depicted in Figure 2d–f, during a 10 µs irradiation period at 1000 kHz, the temperature of the Invar alloy rose from 300 K to 1070 K. With longer inter-pulse intervals (lower repetition rate), the temperature rise diminished, reaching 970 K at 500 kHz and only 750 K at 200 kHz. The shorter 1 µs inter-pulse interval at 1000 kHz enables efficient heat accumulation through successive pulses, facilitating effective energy transfer and sustained thermal effects. Conversely, the longer 5 µs interval at 200 kHz significantly constrains the cumulative heating effect, resulting in lower final accumulated heat.

These findings demonstrate that, under constant auxiliary conditions, parameter adjustments (repetition rate and pulse energy) exert critical influence over the thermal processes during processing. By strategically coordinating the selection of repetition rate and pulse energy, the dynamics of thermal ablation can be precisely regulated, thereby enabling control over both the extent of sample damage and the melt volume. This parameter control strategy will prove instrumental in the subsequent quest to identify the optimal single-spot welding parameters.

### 3.2. Ablation Threshold of Sapphire and Invar Alloy at Different Repetition Rates

**Figure 3:**
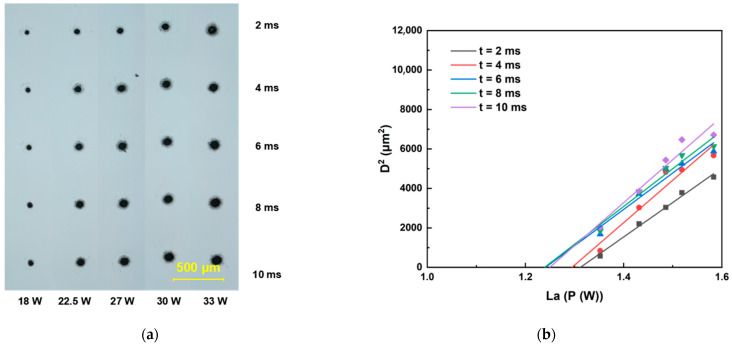
Ablation threshold determination results for sapphire. (**a**) Morphology of ablation craters produced by femtosecond laser irradiation at 500 kHz. (**b**) Corresponding ablation threshold fitting curves for 500 kHz.

**Figure 4:**
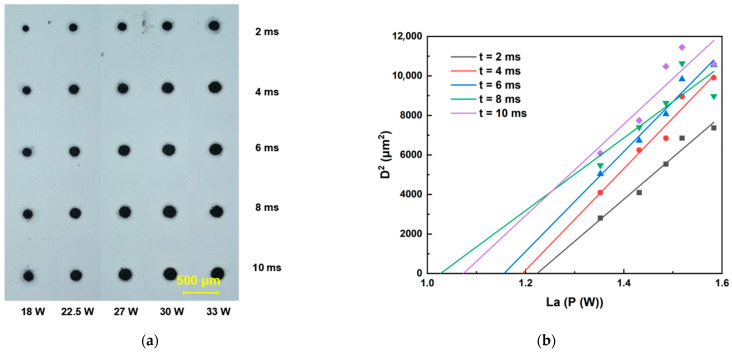
Ablation threshold determination results for sapphire. (**a**) Morphology of ablation craters produced by femtosecond laser irradiation at 200 kHz. (**b**) Corresponding ablation threshold fitting curves for 200 kHz.

**Figure 5:**
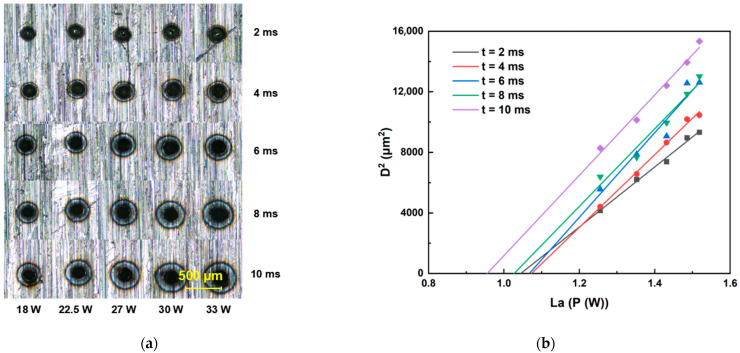
Ablation threshold determination results for Invar alloy. (**a**) Morphology of ablation craters produced by femtosecond laser irradiation at 1000 kHz. (**b**) Corresponding ablation threshold fitting curves for 1000 kHz.

**Figure 6:**
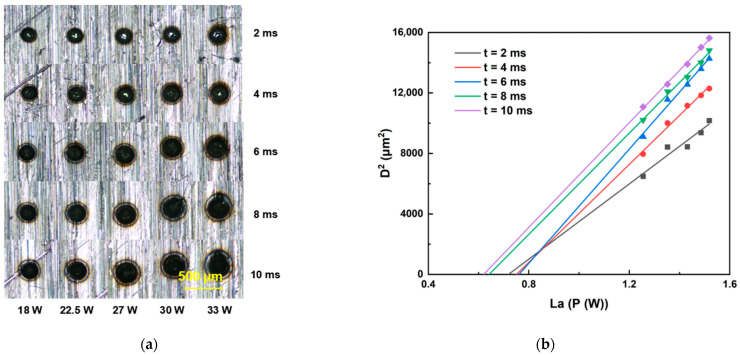
Ablation threshold determination results for Invar alloy. (**a**) Morphology of ablation craters produced by femtosecond laser irradiation at 500 kHz. (**b**) Corresponding ablation threshold fitting curves for 500 kHz.

**Figure 7:**
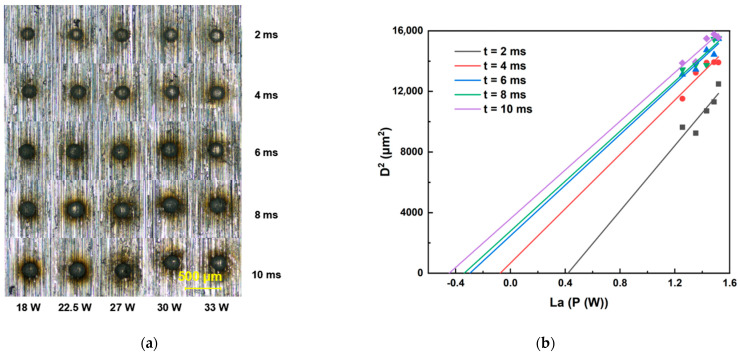
Ablation threshold determination results for Invar alloy. (**a**) Morphology of ablation craters produced by femtosecond laser irradiation at 200 kHz. (**b**) Corresponding ablation threshold fitting curves for 200 kHz.

**Figure 8:**
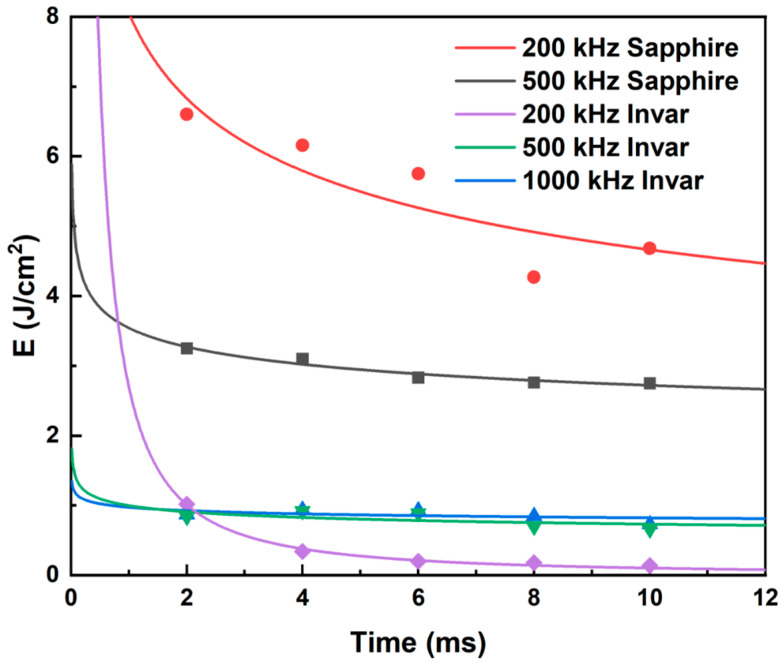
Multipulse ablation threshold fitting results of sapphire and Invar alloy for different repetition rates.

This section details the determination of ablation thresholds for sapphire and Invar alloy using a galvanometer scanner optical path system. The femtosecond laser ablation duration was set within the range of 2 ms to 10 ms. For each fixed ablation duration, the laser energy was incrementally increased to ablate both sapphire and Invar alloy samples. All ablation crater diameters were quantified using an optical microscope. Threshold determinations were performed at repetition rates of 200, 500, and 1000 kHz, with the raw data presented in Figure 3, Figure 4, Figure 5, Figure 6 and Figure 7. Linear regression analysis was applied to the square of the ablation crater diameter (D^2^) versus the logarithm of the average power with respect to 10 (La). The results reveal significant correlation coefficients under the same pulse count conditions. The x-intercepts of these fitted regression lines correspond to the experimentally determined ablation thresholds for their respective pulse counts. Finally, the ablation threshold curve is shown in Figure 8. By integrating experimental methods with numerical analysis, this section systematically investigates variation in material ablation thresholds at different repetition rates. This approach aims to elucidate the thermal effect mechanisms inherent in ultrafast laser processing and provides critical data references for subsequent single-spot welding experiments.

The ablation thresholds obtained using the D^2^ method were compiled, and the ablation threshold curves were plotted. As shown in Figure 8, sapphire exhibits distinct threshold characteristics: at repetition rates of 200 kHz and 500 kHz (when the repetition rate was 1000 kHz, femtosecond laser could only generate ablation craters on the sapphire surface when the laser power reached 30 W), the ablation threshold decreases with increasing pulse count and subsequently stabilizes. Notably, the ablation threshold at 200 kHz is significantly higher than that at 500 kHz. This behavior originates from the differences in pulse energy accumulation caused by the varying inter-pulse time intervals. At higher repetition rates, the increase in the ablation threshold is associated with enhanced thermal effects, which enlarge the ablation crater dimensions. For Invar alloy, the ablation threshold curves at different repetition rates also show an initial decrease, followed by stabilization as the pulse count increases. However, the final stabilized ablation threshold does not progressively decrease with increasing repetition rate. Instead, the lowest stabilized ablation threshold occurs at 200 kHz, while the thresholds at 500 kHz and 1000 kHz are comparable.

Analysis combined with the simulation results reveals that, when the irradiation duration is below 2 ms, the ablation threshold decreases with increasing repetition rate. This observation is consistent with the simulation results, which indicate that higher repetition frequencies (shorter inter-pulse intervals) promote greater pulse energy accumulation. However, under prolonged irradiation duration, the ablation threshold curve at 200 kHz exhibits a more pronounced decreasing trend compared to the curves at 500 kHz and 1000 kHz, resulting in the ablation threshold at 200 kHz gradually falling below those at 500 kHz and 1000 kHz. This phenomenon likely stems from the significantly higher absorption coefficient of Invar alloy for 1030 nm laser radiation. Consequently, a substantial portion of the laser energy is deposited within the Invar alloy. As the laser ablation time extends, the accumulated laser energy in the Invar alloy progressively increases. Under these conditions, the dominant influence of inter-pulse thermal accumulation diminishes, and the higher single-pulse energy at the lower repetition rate (200 kHz) becomes more effective at enlarging the ablation crater area. Concurrently, once the ablation duration exceeds 6 ms, the area of the ablation crater formed by the 200 kHz femtosecond laser irradiation tends to saturate. This saturation effect will also contribute to a reduction in the ablation threshold determined via the D^2^ method.

Comparing the ablation thresholds of Invar alloy and sapphire reveals that, under identical repetition rates, sapphire exhibits a significantly higher ablation threshold than Invar alloy. This implies that, during the laser welding process of sapphire to Invar alloy, the laser energy must at least approach sapphire’s ablation threshold to achieve robust welding strength. In subsequent single-spot welding experiments, laser energy parameters will be selected based on sapphire’s ablation threshold, with the aim of identifying the optimal welding parameters.

### 3.3. Single-Spot Welding Results

Based on the ablation threshold determination results, this section will select laser power parameters for performing single-spot welding. The shear strength of the welded sapphire/Invar alloy samples was measured using a dynamometer. This allowed evaluation of variation in the shear strength of the welded samples under different laser parameters, including energy and repetition rate. Concurrently, the fracture surfaces of the sheared samples were examined using optical microscopy to identify the underlying causes for the observed differences in shear strength.

**Figure 9:**
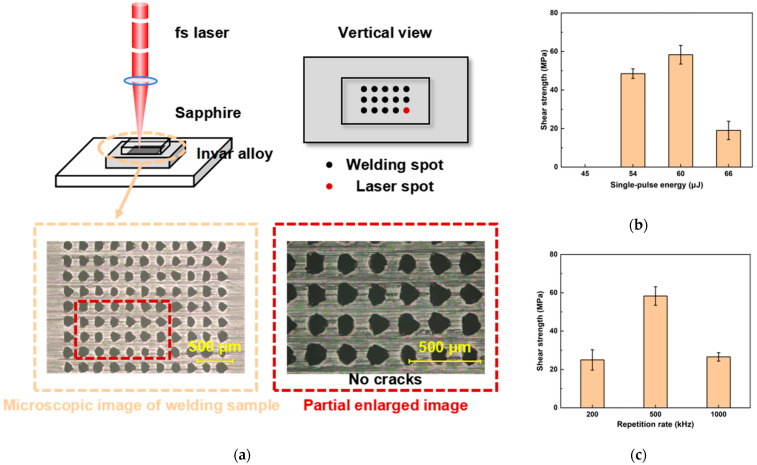
Experimental results of ultrafast laser single-spot welding of the sapphire/Invar alloy. (**a**) Schematic representation of the single-spot welding process; (**b**) measured shear strength at different pulse energies; (**c**) measured shear strength at different repetition rates.

Figure 9a shows a schematic diagram of the single-spot welding process. Utilizing the galvanometer scanner system, the femtosecond laser beam is precisely controlled to move along the interface between sapphire and Invar alloy, creating an arrayed distribution of processing points. A total of 121 points are processed, with a processing time of 10 ms per point and an inter-point spacing of 0.2 mm. Samples welded using the femtosecond laser at a repetition rate of 500 kHz are tested, and the resulting shear strength of the welded samples as a function of pulse energy is presented in Figure 9b. At a single-pulse energy of 45 μJ, bonding between sapphire and Invar alloy occurred, but the shear strength was too low to be detected by the tensiometer. The corresponding energy density was 3.58 J/cm^2^, which was above the damage threshold of sapphire (2.75 J/cm^2^) for a 10 ms irradiation duration. At this energy level, the femtosecond laser induces melting in the sapphire, but the limited melt volume prevents formation of sufficient contact with the Invar alloy, resulting in low shear strength. Subsequently, the shear strength initially increases, but then decreases with further increases in pulse energy. This trend is due to the increasing melt volume in the sapphire at higher pulse energies, leading to a larger contact area with the Invar alloy and consequently enhancing shear strength. However, at a single-pulse energy of 66 μJ, excessive internal damage occurred within the sapphire, causing the shear strength of the welded sample to decrease.

**Figure 10:**
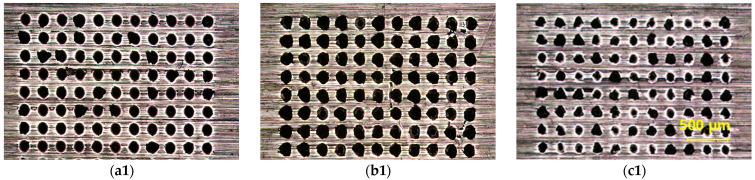

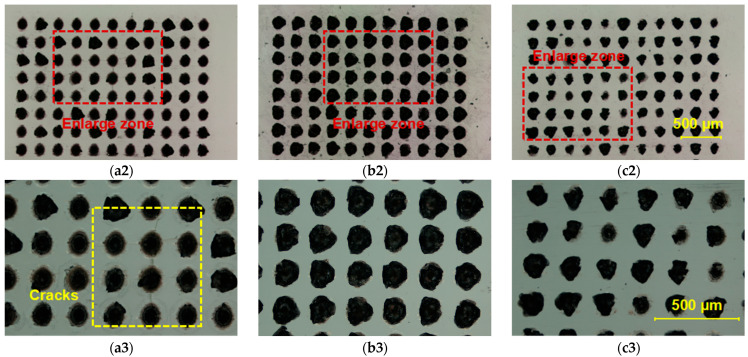
Fracture surface morphology of sapphire and Invar alloy after tensile testing of welded samples processed at different repetition rates: (**a1**) 200 kHz Invar alloy; (**a2**) 200 kHz sapphire; (**b1**) 500 kHz Invar alloy; (**b2**) 500 kHz sapphire; (**c1**) 1000 kHz Invar alloy; (**c2**) 1000 kHz sapphire. (**a3**–**c3**) are partial enlarged views of (**a2**–**c2**).

Figure 9c presents the shear strength of samples welded using a femtosecond laser at different repetition rates. The laser power was held 30W, where the resulting energy density exceeded the damage threshold of sapphire for a 10 ms irradiation duration at each respective rate. Figure 9c revealed that the highest shear strength (63.37 MPa) was achieved at a repetition rate of 500 kHz, which was similar to the highest strength of 69 MPa obtained by high-temperature sintering brazing [12]. When the rate was changed to 200 kHz or 1000 kHz, the shear strength of the welded samples decreased significantly, to approximately 25 MPa. Figure 10 displays microscopic surface morphologies of the fractured sapphire and Invar alloy interfaces. The Invar alloy surface showed residual molten sapphire adhering to it, while corresponding defects were observable on the sapphire surface. Comparing the residual sapphire area on the Invar alloy surfaces across repetition rates revealed that the area at 1000 kHz was smaller than that at 500 kHz. This reduced contact area explains the lower shear strength at 1000 kHz compared to 500 kHz. At 200 kHz, the residual sapphire area on the Invar alloy surface was comparable to that at 500 kHz. However, cracks developed on the sapphire surface at 200 kHz. These cracks significantly reduce the shear strength of sapphire itself, accounting for the reduced strength observed at 200 kHz relative to 500 kHz.

Analysis of the above experimental results reveals that both single-pulse energy and repetition rate significantly impact the strength of welded samples. Excessively low pulse energy results in an insufficient molten area of sapphire, or even fails to melt it, preventing adequate contact between the sapphire and Invar alloy and leading to unacceptably low shear strength in the welded samples. Conversely, excessively high pulse energy causes severe internal damage within the sapphire, which also adversely affects the shear strength of the welded samples. The repetition rate influences the energy deposition efficiency of the femtosecond laser on the sample. Too low a repetition rate reduces energy deposition efficiency and simultaneously leads to excessively high single-pulse energy. While a higher repetition rate improves energy deposition efficiency, it also results in insufficient single-pulse energy.

### 3.4. Mechanism of Thermal Effect Reduction

The maximum shear strength achieved by single-spot welded samples reached 63.37 MPa, a level difficult to achieve with conventional scanning welding. To investigate the underlying reasons, this subsection describes both single-spot welding with reduced spot spacing (which we refer to as dense single-spot welding) and traditional scanning welding on sapphire/Invar alloy using optimized parameters. Post-welding samples underwent strength testing and microscopic characterization. Through comparative analysis, the advantages of single-spot welding will be elucidated.

**Figure 11:**
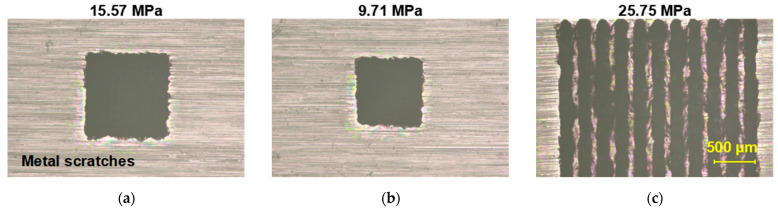

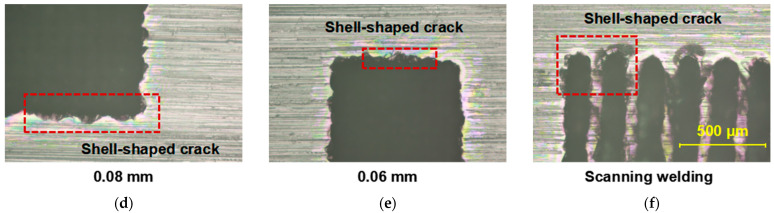
Experimental results of dense single-spot welding and traditional scanning welding. (**a**–**c**) Microscopic morphology of welding areas under different working conditions. (**d**–**f**) Partial enlarged view of welding area under various working conditions.

Figure 11a–c present micrographs of welded samples processed via dense single-spot welding and traditional scanning welding. The shear strengths of samples with spot spacings of 0.08 mm and 0.06 mm were 15.57 MPa and 9.71 MPa, respectively, while the scanning-welded sample exhibited a shear strength of 25.75 MPa. All values are lower than that of standard spacing (0.2 mm) single-spot welding.

Figure 11d–f show zoomed-in views of the regions in Figure 11a–c. These images reveal varying degrees of sapphire damage at the periphery of the welded zones under all three processing conditions. Both dense single-spot welding and scanning welding demonstrate higher spatial energy concentration compared to standard single-spot welding, resulting in pronounced thermal effects that caused excessive damage to the sapphire substrate.

**Figure 12:**
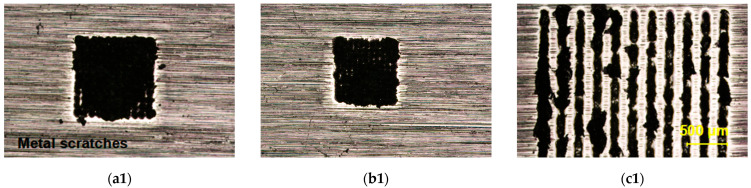

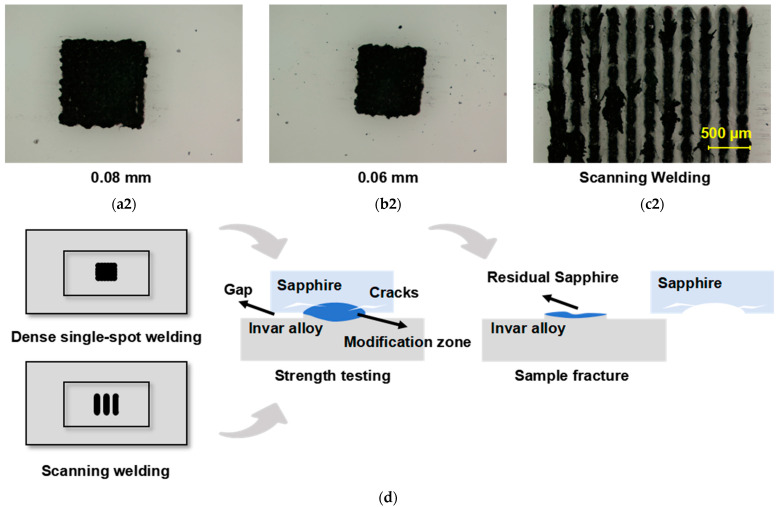
Comparative fracture surface morphology of sapphire and Invar alloy after tensile testing of dense single-spot welding and traditional scanning welding: (**a1**) 0.08 mm Invar alloy; (**a2**) 0.08 mm sapphire; (**b1**) 0.06 mm Invar alloy; (**b2**) 0.06 mm sapphire; (**c1**) scanning welding Invar alloy; (**c2**) scanning welding sapphire. (**d**) Schematic diagram of the mechanism of welding strength reduction.

Figure 12 presents the microscopic fracture surface morphologies of sapphire and Invar alloy for samples processed under three conditions. Figure 12(a1,b1) reveal that reducing spot spacing enhances spatial energy concentration, thereby strengthening the welded joint. However, this intensified energy concentration simultaneously exacerbates sapphire damage. The presence of extensive molten sapphire residue on the Invar alloy surface indicates that fracture primarily occurred within the sapphire bulk, resulting in significantly lower shear strength compared to standard spacing welds. Conversely, Figure 12(c1) demonstrates that scanning welding’s spatially distributed thermal accumulation effectively expands the sapphire/Invar alloy contact area, improving joint strength. While fractures still predominantly initiate within sapphire, the substantially reduced internal damage (relative to dense single-spot welding) enables scanning welded samples to maintain moderate shear strength. Figure 12d illustrates the mechanism of the decrease in welding strength.

## 4. Conclusions

This study combines numerical simulation and process experiments for a systematic investigation. By examining thermal accumulation effects, ablation thresholds, and welding methodologies, we aim to mitigate thermal effects during glass/metal welding. The findings provide methodological guidance for optimizing ultrafast laser welding of glass and metal under non-optical contact conditions.

(1) This study establishes a femtosecond laser ablation model for Invar alloy to investigate thermal accumulation phenomena during the ablation process. The simulations ultimately reveal that, when the total laser energy remains constant, higher repetition frequencies result in greater heat accumulation within the Invar alloy. Under femtosecond laser irradiation at a 1000 kHz repetition rate and 30 μJ pulse energy, the temperature of Invar alloy elevates from 300 K to 1070 K within 10 μs.

(2) Femtosecond laser multipulse ablation experiments were conducted on sapphire and Invar alloy to investigate their ablation thresholds at different repetition frequencies. Ablation crater data for each rate were processed using the D^2^ method for mathematical analysis, ultimately yielding ablation threshold curves for both materials across repetition frequencies. Across all tested repetition rates, the ablation threshold of sapphire consistently and significantly exceeds that of Invar alloy.

(3) Building upon model simulation results and ablation threshold curves, we conducted a study of femtosecond laser single-spot welding of sapphire and Invar alloy, investigating the influence of single-pulse energy and repetition rate on the welding strength of sapphire–Invar joints. Finally, sapphire and Invar alloy are welded using a femtosecond laser with a single-pulse energy of 60 μJ and a repetition rate of 500 kHz, achieving a shear strength of 63.37 MPa for the welded samples.

(4) We conducted dense single-spot welding and traditional scanning welding on sapphire and Invar alloy based on optimal single-spot welding parameters. By analyzing defects in the weld cross-sections at the microscopic level, we elucidated the mechanism responsible for the strength degradation. This analysis further demonstrates the superiority of single-spot welding technology over conventional methods in mitigating thermal effects.

Although femtosecond laser single-spot welding effectively reduces thermal effects and enhances joint quality in dissimilar materials welding, challenges remain in processing time and area requirements. These limitations hinder industrial adoption and necessitate further research. We envision this technology providing a novel approach for dissimilar material joining, with foreseeable applications in microelectronics packaging, medical device fabrication, and sensor manufacturing, where it could contribute substantially.

## Figures and Tables

**Table 1:** Invar alloy properties for the simulation [54,56,58].

Property	Symbol	Value (Alumina)
Electronic heat capacity coefficient	C	$545\;\left(J\cdot m^{-3}\cdot K^{2}\right)$
Free electron density	$N_{e}$	$5.4\;\times \;10^{16}\;\left({\mathrm{cm}}^{-3}\right)$
Boltzmann constant	$k_{B}$	$1.380649\;\times \;10^{-23}\;\left(J\cdot K^{-1}\right)$
Fermi temperature	$T_{f}\;$	$81,353\;\left(K\right)$
Lattice heat capacity	$C_{l}\;$	$4.18\;\times {\;10}^{6}\;\left(J\cdot m^{-3}\cdot K^{-1}\right)$
Electron–lattice coupling coefficient	G	$3.18\;\times \;10^{17}\;\left(W\cdot m^{-3}{\cdot K}^{-1}\right)$
Electron–lattice coupling coefficient of iron	$G_{\mathrm{Fe}}$	$3\;\times \;10^{17}\;\left(W\cdot m^{-3}{\cdot K}^{-1}\right)$
Electron–lattice coupling coefficient of nickel	$G_{\mathrm{Ni}}$	$3.6\;\times \;10^{17}\;\left(W\cdot m^{-3}{\cdot K}^{-1}\right)$
Material absorption coefficient	$\delta_{0}$	$9\;\times \;10^{6}\;\left(m^{-1}\right)$
Wavelength	$\lambda_{0}$	$1030\;\left(\mathrm{nm}\right)$
Spot radius	$r_{0}$	$20\;\left(\mu m\right)$
Invar alloy reflectivity	R	0.3

**Table 2:** The laser parameters for the experiment.

Parameter	Range
Pulse energy, *E* (µJ)	18 ≤ *E* ≤ 191.5
Puls repetition rate, *f* (kHz)	200 ≤ *f* ≤ 1000
Welding time, *t_on_* (ms)	2 ≤ *t_on_* ≤ 10

## Data Availability

The original contributions presented in the study are included in the article; further inquiries can be directed to the corresponding author.
